# Supplementary material for: Post-Exercise Whey Protein Supplementation: Effects on IGF-1, Strength, and Body Composition in Pre-Menopausal Women, a Randomised Controlled Trial
Source: Nutrients. 2025 Jun 18;17(12):2033. doi: 10.3390/nu17122033 (PMC12196337; doi:10.3390/nu17122033)
Supplement: Supplementary file 1 [file nutrients-17-02033-s001.zip › nutrients-3653571-supplementary.pdf]

Table S1. Dietary information for the control group (CON) and moderate protein group (PRO) used in statistical analyses taken from baseline (T0), week four (T4), eight (T8) and twelve (T12).

	T0		T4		T8		T12		Mean difference			Significance		
	CON (*n* = 11)	PRO (*n* = 13)	CON (*n* = 11)	PRO (*n* = 13)	CON (*n* = 11)	PRO (*n* = 13)	CON (*n* = 11)	PRO (*n* = 11)	T4	T8	T12	Group	Time	Interaction
Energy (kJ)	7742 (1361)	6868 (1743)	7924 (1463)	7205 (1324)	7622 (1189)	6861 (1385)	8061 (1475)	7616 (1552)	155 (- 1118, 1428)	112 (- 1161, 1386)	434 (- 871, 1739)	0.131	0.251	0.925
Carbohydrate (g)	176 (65)	157 (56)	197 (54)	159 (58)	188 (54)	158 (59)	185 (48)	176 (54)	- 18.7 (- 55.9, 18.5)	- 10.6 (- 47.8, 26.6)	12.5 (- 25.6, 50.7)	0.248	0.426	0.397
Fat (g)	83.2 (18.8)	75.0 (30.3)	78.6 (23.7)	78.4 (18.7)	78.4 (27.8)	69.6 (18.1)	90.7 (26.5)	79.8 (21.1)	3.0 (- 17.9, 23.8)	- 0.5 (- 21.4, 20.3)	- 3.0 (- 24.3, 18.4)	0.256	0.194	0.957
Protein (g)	78.9 (12.3)	70.1 (20.4)	80.7 (16.9)	95.7 (14.4)	84.5 (17.8)	85.3 (15.3)	86.7 (29.7)	90.5 (24.4)	23.8 (- 7.7, 40.0)	9.7 (- 6.5, 25.8)	12.8 (- 3.8, 29.4)	0.621	0.003	0.039*
Protein (g/kg BW)	1.06 (0.17)	1.18 (0.40)	1.09 (0.28)	1.61 (0.41)	1.14 (0.29)	1.42 (0.33)	1.16 (0.26)	1.49 (0.44)	0.40 (0.16, 0.64)	0.16 (- 0.80, 0.41)	0.22 (- 0.03, 0.47)	0.012	0.002	0.017*

Data presented as observed means (SD) for respective timepoints. Mean difference (95% CI) between intervention and control group and significance data taken from linear mixed-effects model analyses. * Indicates a significant (p < 0.05) group × time interaction effect. SD = standard deviation, n = number of participants, kJ = kilojoule, g = gram, g/kg BW = gram/kilogram of body weight.

Table S2. Unadjusted and adjusted models for IGF-1, body composition and strength outcomes.

Variable	Estimate	Std. Error	Significance	95% Confidence Interval Lower Bound	Upper Bound	ηp²
UNADJUSTED						
**Total IGF-1 (µg/L)**	3.988	10.750	0.714	-18.151	26.128	
ADJUSTED						
**Total IGF-1 (µg/L)**	1.380	13.555	0.920	-26.660	29.420	
Age	-4.511	0.949	< 0.001	-6.480	-2.542	0.51
Protein (g/kg lean)	-2.471	13.401	0.855	-29.665	24.723	0.001
UNADJUSTED						
**Body mass (kg)**	-0.275	0.626	0.662	-1.523	0.973	
UNADJUSTED						
**BMI (kg/m2)**	-0.733	0.204	0.721	-0.480	0.334	
UNADJUSTED						
**Total lean mass (kg)**	0.289	0.484	0.556	-1.289	0.710	
ADJUSTED						
**Total lean mass (kg)**	-0.163	0.506	0.750	-1.221	0.895	
IGF-1 (µg/L)	11.683	7.972	0.153	-4.590	27.956	0.07
Fat intake (g)	-15.036	10.719	0.175	-37.331	7.258	0.09
UNADJUSTED						
**Upper body lean mass (kg)**	0.169	0.309	0.590	-0.807	0.469	
ADJUSTED						
**Upper body lean mass (kg)**	-0.080	0.344	0.820	-0.800	0.641	
IGF-1 (µg/L)	5.467	5.213	0.302	-5.130	16.064	0.03
Fat intake (g)	-6.376	7.242	0.388	-21.405	8.654	0.03
UNADJUSTED						
**Trunk lean mass (kg)**	-0.174	0.302	0.570	-0.798	0.449	
ADJUSTED						
**Trunk lean mass (kg)**	-0.072	0.338	0.834	-0.779	0.635	
IGF-1 (µg/L)	4.475	4.776	0.355	-5.190	14.145	0.02
Fat intake (g)	-4.387	7.006	0.537	-18.880	10.106	0.02
UNADJUSTED						
**Arm lean mass (kg)**	0.003	0.060	0.958	-0.121	0.128	
ADJUSTED						

**Arm lean mass (kg)**	-0.007	0.067	0.913	-0.147	0.132	
IGF-1 (µg/L)	1.501	1.077	0.174	-0.702	3.705	0.06
Fat intake (g)	-1.250	1.423	0.390	-4.211	1.712	0.04
UNADJUSTED						
**Leg lean mass (kg)**	-0.118	0.206	0.571	-0.545	0.307	
ADJUSTED						
**Leg lean mass (kg)**	-0.089	0.199	0.659	-0.505	0.327	
IGF-1 (µg/L)	6.045	3.177	0.067	-0.451	12.542	0.11
Fat intake (g)	-8.369	4.221	0.061	-17.156	0.419	0.16
UNADJUSTED						
**Total fat mass (kg)**	-0.020	0.661	0.976	-1.385	1.344	
ADJUSTED						
**Total fat mass (kg)**	-0.317	0.610	0.609	-1.593	0.958	
IGF-1 (µg/L)	-0.602	8.841	0.946	-18.791	17.587	0.0001
Protein intake (g)	14.228	17.467	0.425	-22.180	50.636	0.03
UNADJUSTED						
**Upper body fat mass (kg)**	0.005	0.453	0.991	-0.931	0.941	
ADJUSTED						
**Upper body fat mass (kg)**	-0.089	0.396	0.825	-0.916	0.739	
IGF-1 (µg/L)	-3.915	5.778	0.504	-15.820	7.990	0.02
Protein intake (g)	6.398	11.340	0.579	-17.252	30.048	0.02
UNADJUSTED						
**Trunk fat mass (kg)**	-0.020	0.415	0.962	-0.877	0.838	
ADJUSTED						
**Trunk fat mass (kg)**	-0.091	0.317	0.778	-0.753	0.572	
IGF-1 (µg/L)	-3.802	4.639	0.420	-13.366	5.761	0.03
Protein intake (g)	4.964	9.078	0.591	-13.970	23.899	0.01
UNADJUSTED						
**Arm fat mass (kg)**	0.024	0.081	0.765	-0.142	0.191	
ADJUSTED						
**Arm fat mass (kg)**	-0.003	0.109	0.980	-0.231	0.225	
IGF-1 (µg/L)	0.251	1.484	0.867	-2.776	3.278	0.0009
Protein intake (g)	1.656	3.091	0.598	-4.768	8.080	0.01
UNADJUSTED						
**Leg fat mass (kg)**	-0.030	0.240	0.903	-0.524	0.465	
ADJUSTED						

**Leg fat mass (kg)**	-0.229	0.257	0.384	-0.765	0.308	
IGF-1 (μg/L)	3.137	3.628	0.395	-4.294	10.569	0.03
Protein intake (g)	7.592	7.325	0.312	-7.647	22.833	0.05
UNADJUSTED						
**Visceral adipose tissue (g)**	-5.250	38.070	0.891	-83.800	73.300	
UNADJUSTED						
**Bone mineral density (g/cm2)**	0.013	0.010	0.176	-0.006	0.033	
UNADJUSTED						
**Body fat (%)**	-0.090	0.614	0.885	-1.350	1.178	
UNADJUSTED						
**3RM Chest press (kg)**	-0.711	0.798	0.381	-2.350	0.932	
ADJUSTED						
**3RM Chest press (kg)**	-0.274	0.974	0.781	-2.290	1.740	
IGF-1 (μg/L)	0.007	0.008	0.433	-0.010	0.023	0.02
Protein intake (g)	-0.028	0.024	0.240	-0.776	0.020	0.04
Upper body lean mass (kg)	0.519	0.198	0.015	0.111	0.927	0.22
UNADJUSTED						
**3RM Row (kg)**	-0.398	0.789	0.618	-2.023	1.227	
ADJUSTED						
**3RM Row (kg)**	-0.674	0.968	0.493	-2.678	1.329	
IGF-1 (μg/L)	0.001	0.008	0.860	-0.015	0.018	0.001
Protein intake (g)	0.018	0.024	0.447	-0.030	0.067	0.02
Upper body lean mass (kg)	0.745	0.193	< 0.001	0.347	1.140	0.37
UNADJUSTED						
**3RM Shoulder press (kg)**	-0.733	0.921	0.433	-2.630	1.160	
ADJUSTED						
**3RM Shoulder press (kg)**	-0.671	1.090	0.544	-2.923	1.581	
IGF-1 (μg/L)	0.001	0.008	0.880	-0.014	0.017	0.0008
Protein intake (g)	-0.016	0.025	0.522	-0.066	0.034	0.01
Upper body lean mass (kg)	0.606	0.176	0.002	0.242	0.969	0.34

Estimates are taken from interaction between group*time within the model.

Table S3. Observed results (mean ± SD) of 3RM every four weeks and training volume over twelve weeks in both the control group (CON) and the moderate protein group (PRO).

	T0		T4		T8		T12	
	CON (*n* = 12)	PRO (*n* = 15)	CON (*n* = 12)	PRO (*n* = 15)	CON (*n* = 12)	PRO (*n* = 15)	CON (*n* = 12)	PRO (*n* = 15)
Three repetition-max (3RM)								
Chest press (kg)	14.2 (2.7)	13.6 (3.2)	17.1 (3.2)	16.3 (3.0)	19.3 (3.8)	18.4 (3.3)	21.2 (4.2)	19.8 (3.7)
Single-arm row (kg)	18.1 (2.8)	16.7 (3.8)	21.3 (3.3)	20.1 (4.1)	23.5 (3.6)	22.0 (4.2)	25.4 (3.9)	23.6 (4.2)
Shoulder press (kg)	11.8 (2.6)	11.9 (3.2)	14.8 (3.5)	14.4 (3.5)	17.2 (4.0)	16.4 (3.4)	18.8 (4.3)	18.1 (3.7)
Total training volume								
Chest press (kg)							7998 (1764)	7142 (1366)
Single-arm row (kg)							9645 (2015)	8557 (1636)
Shoulder press (kg)							6687 (1848)	6175 (1451)

Data presented as mean (SD). kg = kilogram.

Table S4. Sensitivity analyses for all outcome variables

				95% Confidence Interval	
**Variable**	**Estimate**	**Std. Error**	**Significance**	**Lower Bound**	**Upper Bound**
**Total IGF-1 (μg/L)**	-33.711	14.6398	0.033	-64.3199	-3.1024
Age (y)	-4.7645	0.881752	< 0.001	-6.6	-2.929
Protein intake (g/kg lean)	2.305499	12.3493	0.853	-22.7744	27.385
**Body mass (kg)**	-0.793	0.882	0.371	-2.551	0.965
**BMI (kg/m²)**	-0.0068	0.2803	0.981	-0.5657	0.5521
**Total lean mass (kg)**	-0.554	0.734	0.459	-2.087	0.980
IGF-1 (μg/L)	8.641	9.149	0.353	-10.091	27.374
Fat intake (g)	-14.356	10.801	0.199	-36.943	8.231
**Upper body lean mass (kg)**	-0.471	0.490	0.348	-1.495	0.552
IGF-1 (μg/L)	3.322	5.905	0.578	-8.713	15.359
Fat intake (g)	-6.108	7.215	0.847	-21.170	8.953
**Trunk lean mass (kg)**	-0.456	0.474	0.347	-1.444	0.532
IGF-1 (μg/L)	2.865	5.343	0.595	-7.969	13.700
Fat intake (g)	-4.144	6.959	0.558	-18.624	10.335
**Arm lean mass (kg)**	0.029	0.096	0.763	-0.171	0.230
IGF-1 (μg/L)	1.731	1.225	0.169	-0.786	4.248
Fat intake (g)	-1.425	1.411	0.325	-4.377	1.525
**Leg lean mass (kg)**	-0.020	0.296	0.946	-0.639	0.599
IGF-1 (μg/L)	5.905	3.712	0.123	-1.700	13.510
Fat intake (g)	-8.096	4.359	0.079	-17.210	1.018
**Total fat mass (kg)**	-0.272	0.795	0.739	-1.389	1.933
IGF-1 (μg/L)	-1.407	10.260	0.892	-22.525	19.709
Protein intake (g)	10.037	11.694	0.401	-14.411	34.486
**Upper body fat mass (kg)**	0.206	0.512	0.692	-0.865	1.276
IGF-1 (μg/L)	-4.341	6.647	0.520	-18.042	9.359
Protein intake (g)	5.163	7.531	0.501	-10.588	20.916
**Trunk fat mass (kg)**	-0.102	0.413	0.808	-0.762	0.966
IGF-1 (μg/L)	-4.350	5.386	0.427	-15.461	6.761
Protein intake (g)	2.949	6.075	0.633	-9.761	15.660
**Arm fat mass (kg)**		0.135	0.421	-0.170	0.392
IGF-1 (μg/L)	0.200	1.618	0.902	-3.094	3.495
Protein intake (g)	2.462	1.985	0.229	-1.672	6.596
**Leg fat mass (kg)**	0.074	0.333	0.835	-0.625	0.766
IGF-1 (μg/L)	2.825	4.210	0.508	-5.806	11.456

Protein intake (g)	4.641	4.907	0.356	-5.602	14.880
**Visceral adipose tissue (g)**	-8.214	53.460	0.879	-118.790	102.360
**Bone mineral density (g/cm^2)**	-0.019	0.013	0.162	-0.047	0.008
**Body fat (%)**	0.279	0.861	0.749	-1.502	2.060
**3RM Chest press (kg)**	-0.468	1.191	0.699	-2.952	2.016
IGF-1 (µg/L)	0.007	0.009	0.446	-0.011	0.025
Protein intake (g)	-0.020	0.024	0.415	-0.068	0.029
Upper body lean mass (kg)	0.585	0.188	0.005	0.197	0.974
**3RM Row (kg)**	-0.310	1.242	0.806	-2.897	2.277
IGF-1 (µg/L)	0.009	0.008	0.918	-0.016	0.018
Protein intake (g)	0.017	0.024	0.486	-0.031	0.065
Upper body lean mass (kg)	0.891	0.176	< 0.001	0.527	1.254
**3RM Shoulder press (kg)**	0.743	1.377	0.596	-2.128	3.614
IGF-1 (µg/L)	0.003	0.008	0.701	-0.014	0.020
Protein intake (g)	-0.016	0.025	0.528	-0.066	0.035
Upper body lean mass (kg)	0.617	0.170	0.002	0.263	0.970

Estimates are taken from interaction between group*time within the model.
